# Reply to: Extracting Kondo temperature of strongly-correlated systems from the inverse local magnetic susceptibility

**DOI:** 10.1038/s41467-021-21643-0

**Published:** 2021-03-04

**Authors:** Xiaoyu Deng, Katharina M. Stadler, Kristjan Haule, Seung-Sup B. Lee, Andreas Weichselbaum, Jan von Delft, Gabriel Kotliar

**Affiliations:** 1 Department of Physics and Astronomy, Rutgers University, Piscataway, NJ USA; 2 Physics Department, Arnold Sommerfeld Center for Theoretical Physics and Center for NanoScience, Ludwig-Maximilians-Universitat München, München, Germany; 3 Condensed Matter Physics and Materials Science Department, Brookhaven National Laboratory, Upton, NY USA

**Keywords:** Electronic structure, Electronic properties and materials, Theoretical physics

**Replying to** A. A. Katanin *Nature Communications* 10.1038/s41467-021-21641-2 (2021).

In his comment^1^, Katanin reanalyzes our LDA + DMFT results^2^ for the temperature-dependent static local spin susceptibility of Sr_2_RuO_4_ and V_2_O_3_ fitting them to a Curie–Weiss (CW) form, $\chi \left( T \right) \simeq a/\left( {T + \theta } \right)$. Invoking Wilson’s analysis^3^ of the impurity susceptibility of the spin-½ one-channel Kondo model (1CKM) in the wide-band limit, he extracts spin Kondo temperatures using $T_{{{\mathrm{K}}}} = \theta /\sqrt 2 ,$ obtaining *T*_K_ = 350 K and 100 K for Sr_2_RuO_4_ and V_2_O_3_, respectively. Noting that these are significantly smaller than the scales $T_{{{{\mathrm{sp}}}}}^{{{{\mathrm{onset}}}}}$ = 2300 K and 1000 K reported in ref. ^2^, he argues that our $T_{{{{\mathrm{sp}}}}}^{{{{\mathrm{onset}}}}}$ scales “do not characterize the screening process”.

We welcome Katanin’s use of our data. However, his implication that our $T_{{{{\mathrm{sp}}}}}^{{{{\mathrm{onset}}}}}$ was intended to fully characterize the screening process is misleading. Our work uses the full susceptibility vs. temperature curve to describe properly spin screening, not just a single number. Furthermore, our $T_{{{{\mathrm{sp}}}}}^{{{{\mathrm{onset}}}}}$ was defined to characterize the high-temperature onset of spin screening, whereas his *T*_K_ characterizes the CW regime found at intermediate (i.e., lower) temperatures. The fact that *T*_K_ is much smaller than $T_{{{{\mathrm{sp}}}}}^{{{{\mathrm{onset}}}}}$ is therefore not surprising but natural.

We agree with Katanin that, for Hund metals in general and Sr_2_RuO_4_ in particular, it is reasonable to approximate $\chi \left( T \right)$, using results of a Kondo impurity which features a CW law at intermediate temperatures. (In the Supplementary material we analyze $\chi \left( T \right)$ taken data from DMFT studies of the model Hund system used in ref. ^2^.) However, this was already well known. For Sr_2_RuO_4_, a comparison to the exact solution of a (fully screened) spin-1 Kondo model impurity model was carried out in the inset of Fig. 3a of ref. ^4^ (ref. 17 of ref. ^2^), reproduced as Fig. 1(left) below, and a CW fit of that data was published in Fig. 2a of ref. ^5^ (cited as ref. 5 of ref. ^2^). We reproduce it as Fig. 1(right) below. Since Sr_2_RuO_4_ and V_2_O_3_ have an atomic ground state configuration spin closer to 1 than ½, the use of a (fully screened) spin-1 Kondo model is more reasonable. Furthermore, when interpreting LDA+DMFT results, it is preferable to use definitions of the Kondo scale that rely on the low-temperature portion of the susceptibility curve, as was done in refs. ^4,10^, as opposed to the high-temperature portion as in Katanin’s proposal to characterize spin screening. We elaborate on these points and propose a simple way to characterize spin crossovers of Hund metals below.

**Fig. 1:**
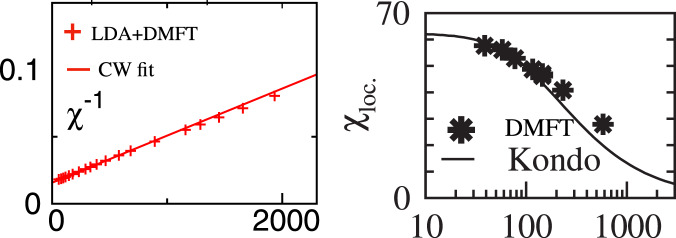
Earlier work comparing Kondo impurity model with LDA+DMFT results for Sr_2_RuO_4_. Left: $1/\chi (T)$ versus *T*, with LDA+DMFT results for Sr_2_RuO_4_ (red symbols) and a Curie–Weiss fit (straight red line) reproduced from the inset of Fig. 2a of ref. ^5^. Right: Bethe-Ansatz results for the spin-1,2-channel Kondo model $\chi \left( T \right)$ vs. *T* with $T_{{{{\mathrm{BA}}}}}$ = 240 K (solid line) in good agreement with the LDA + DMFT results for Sr_2_RuO_4_ (black symbols) reproduced from inset of Fig. 3a of ref. ^4^.

Since Katanin’s comment invokes the 1CKM, we start by summarizing some of its well-established properties^3,6,7^. $\chi \left( T \right)$ exhibits a very broad crossover, from Curie-like high-temperature behavior governed by a local-moment fixed point describing a free spin, to Pauli-like low-temperature behavior governed by a Fermi-liquid fixed point describing a fully screened spin. A proper description of this crossover requires a crossover scaling function, $F\left( {T/T_{{{\mathrm{K}}}}} \right)$ and a crossover scale, the Kondo scale *T*_K_, with $\chi \left( T \right) = F(T/T_{{{\mathrm{K}}}})/T$. Wilson showed that *F*(*x*) is universal under the assumptions of very weak impurity-bath coupling and infinite bandwidth, and computed it numerically. There are multiple ways of defining *T*_K_, evoking the behavior of *F*(x) for either $x \gg 1,$ $x \simeq 1$, or $x \ll 1$, yielding *T*_K_ values differing only by factors of order unity. Wilson’s definition of *T*_K_ (adopted by Katanin), denoted *T*_W_ here, evokes the $x \gg 1$ limit. For high temperatures, $T \,\gtrsim\, 16T_{{{\mathrm{W}}}}$, he found $\chi \left( T \right) \simeq 1/(4T)\left[ {1 - 1/{{{\mathrm{ln}}}}(T/T_{{{\mathrm{W}}}}) + O\left( {1/\ln ^3(T/T_{{{\mathrm{W}}}})} \right.} \right]$, with *T*_W_ defined such that the coefficient of $1/\ln ^2(T/T_{{{\mathrm{W}}}})$ vanishes. For intermediate temperatures, $0.5T_{{{\mathrm{W}}}} \, < \, T \, < \, 16T_{{{\mathrm{W}}}}$, his numerical results are well approximated by a CW form, with *a* = 0.17 and $\theta \sim \sqrt 2 T_{{{\mathrm{W}}}}$^3,6^ (as used by Katanin). At zero temperature, Wilson found $\chi \left( 0 \right)\sim 0.103/T_{{{\mathrm{W}}}}$ (Eq. (IX.91) of ref. ^3^). Subsequent Bethe-Ansatz (BA) calculations of the scaling function^6,7^ matched Wilson’s numerical results. Analogous results have been obtained for fully screened Kondo models with higher spins^8,9^.The BA works showed that the curve $\chi (T)$ vs. *T*/*T*_K_ depends on the spin *S*, with $\chi \left( T \right) \propto \left. {S(S + 1)} \right]/(3T)$ for $T/T_{{{\mathrm{K}}}} \gg 1$ and $\chi \left( T \right) \propto S$ for $T/T_{{{\mathrm{K}}}} \ll 1$. The Kondo scales defined in these BA works are independent of spin as in Eq. (21) of ref. ^9^: $T_{{{{\mathrm{BA}}}}} = S/[\pi \chi \left( 0 \right)]$, with $T_{{{{\mathrm{BA}}}}}/T_{{{\mathrm{W}}}} = 1.55$ for *S* = 1/2.

In ref. ^2^, we used a strategy similar to Wilson’s: we identified the regions where the behavior of $\chi _{{{{\mathrm{spin}}}}}\left( T \right)$ and $\chi _{{{{\mathrm{orb}}}}}\left( T \right)$ is governed by atomic physics or Fermi-liquid theory and numerically computed the crossover function bridging them. We defined two scales for the onset and completion of spin screening, $T_{{{{\mathrm{sp}}}}}^{{{{\mathrm{onset}}}}}$ and $T_{{{{\mathrm{sp}}}}}^{{{{\mathrm{cmp}}}}}$ as the temperatures above or below which $\chi _{{{{\mathrm{spin}}}}}\left( T \right)$ shows pure Curie behavior ($\sim 1/T$) or pure Pauli behavior ($\sim {{{\mathrm{const}}}}$), respectively, and similarly $T_{{{{\mathrm{orb}}}}}^{{{{\mathrm{onset}}}}}$ and $T_{{{{\mathrm{orb}}}}}^{{{{\mathrm{cmp}}}}}$ for orbital screening. Our $T_{{{{\mathrm{sp}}}}}^{{{{\mathrm{onset}}}}}$ and $T_{{{{\mathrm{sp}}}}}^{{{{\mathrm{cmp}}}}}$ scales are similar in spirit to Wilson’s 16*T*_W_ and $0.5T_{{{\mathrm{W}}}}$. So even within the 1CKM framework, an extraction of $T_{{{\mathrm{W}}}}$ from our results, using $T_{{{\mathrm{W}}}} \simeq T_{{{{\mathrm{sp}}}}}^{{{{\mathrm{onset}}}}}/16$, would yield $2300\,{{{\mathrm{K}}}}/16 \simeq 140$ K for Sr_2_RuO_4_ and $1000\,{{{\mathrm{K}}}}/16 \simeq 60$ K, and the order of magnitude discrepancy claimed by Katanin disappears.

Contrary to this crude estimate, in ref. ^2^ we did not assume $T_{{{{\mathrm{sp}}}}}^{{{{\mathrm{onset}}}}}$ to be proportional to a single Kondo scale since even for an impurity model without DMFT self-consistency, $T_{{{{\mathrm{sp}}}}}^{{{{\mathrm{onset}}}}}$ is known to be affected by energy scales not present in the wide-band 1CKM (e.g., a finite bandwidth or a finite charging energy), since such scales cut off high-temperature logarithmic corrections [cf. ref. ^10^, Fig. 2b, c]. This is even more important for Mott systems, where the emergence of a quasi-particle resonance with decreasing temperatures affects the bath bandwidth via DMFT self-consistency.

**Fig. 2:**
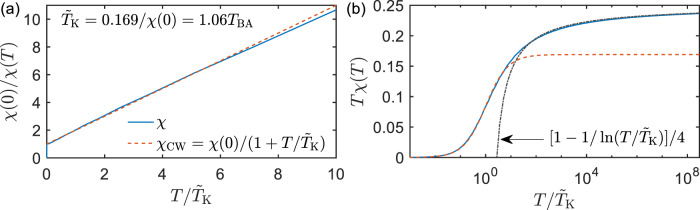
Two representations of the impurity susceptibility $\chi \left( T \right)$ as defined by Wilson^3^ (blue lines), for the 1CKM in the wide-band limit, computed using the numerical renormalization group (NRG). **a** The Curie–Weiss form (red dashed line) works reasonably well for intermediate temperatures, but (**b**) not at all for large temperatures, $T/\tilde T_{{{\mathrm{K}}}} \gg 1$, where logarithmic corrections are large (black dash-dotted line).

In ref. ^2^, we supplemented our LDA+DMFT study of actual materials by DMFT studies of a multi-orbital model Hamiltonian, again computing $\chi (T)$ numerically. We found signatures distinguishing Mottness and Hundness (such as $T_{{{{\mathrm{spin}}}}}^{{{{\mathrm{onset}}}}} \simeq$ $T_{{{{\mathrm{orb}}}}}^{{{{\mathrm{onset}}}}}$ for the former but $T_{{{{\mathrm{spin}}}}}^{{{{\mathrm{onset}}}}} <$ $T_{{{{\mathrm{orb}}}}}^{{{{\mathrm{onset}}}}}$ for the latter) similar to those found in the materials. We defined a Kondo scale $T_{{{{\mathrm{K}}}},{{{\mathrm{spin}}}}}^{{{{\mathrm{dyn}}}}}$ (denoted $T_{{{\mathrm{K}}}}$ in ref. ^2^) through the imaginary part of the $T = 0$ dynamical spin susceptibility, $\chi ^{\prime\prime} \left( {\omega = T_{{{{\mathrm{K}}}},{{{\mathrm{spin}}}}}^{{{{\mathrm{dyn}}}}}} \right)$ = maximal. $T_{{{{\mathrm{K}}}},{{{\mathrm{spin}}}}}^{{{{\mathrm{dyn}}}}}$ characterizes the intermediate region, with $T_{{{{\mathrm{spin}}}}}^{{{{\mathrm{cmp}}}}} < T_{{{{\mathrm{K}}}},{{{\mathrm{spin}}}}}^{{{{\mathrm{dyn}}}}} <$ $T_{{{{\mathrm{spin}}}}}^{{{{\mathrm{onset}}}}}$. It is shown as a red line in Fig. 5b of ref. ^2^, yielding $T_{{{{\mathrm{K}}}},{{{\mathrm{spin}}}}}^{{{{\mathrm{dyn}}}}} = 0.12t = 600\,{{{\mathrm{K}}}}$ for our Hund system H1 mimicking Sr_2_RuO_4_, and $T_{{{{\mathrm{K}}}},{{{\mathrm{spin}}}}}^{{{{\mathrm{dyn}}}}} = 0.04t = 200\,{{{\mathrm{K}}}}$ for our Mott system M1 mimicking V_2_O_3_ (using the conversion factor $t = 5000\,{{{\mathrm{K}}}}$ stated in Fig. 1).

We take Katanin’s comment as an incentive to propose a standardized scheme for extracting a Kondo scale, $\tilde T_{{{\mathrm{K}}}}$, from a computed $\chi \left( T \right)$ curve. Our scheme (i) does not involve a fit to predictions of a specific impurity model, since in general it is unclear which impurity model to compare to, and (ii) uses the $x \le 1$ part of the crossover scaling function, since it is more universal than the $x \gg 1$ part^8–10^; and (iii) reduces to impurity-model results when these are applicable. We propose to define $\tilde T_{{{\mathrm{K}}}}$ through the relation $\chi (\tilde T_{{{\mathrm{K}}}})/\chi (0) = 1/2$. (If $\chi \left( 0 \right)$ is not known but $\chi \left( T \right)$ shows CW-type behavior at intermediate temperatures, $\chi \left( 0 \right)$ can be estimated by linear extrapolation of $1/\chi \left( T \right)$ vs. $T$ to zero temperature.) This definition ensures that $T_{{{{\mathrm{sp}}}}}^{{{{\mathrm{comp}}}}} \, < \, \tilde T_{{{\mathrm{K}}}} \, < \, T_{{{{\mathrm{sp}}}}}^{{{{\mathrm{onset}}}}}$, as it should. For the CW form it yields $\tilde T_{{{\mathrm{K}}}} = \theta$. For the 1CKM, NRG computations (Fig. 2) show that $\tilde T_{{{\mathrm{K}}}} = 0.169/\chi (0) = 1.06T_{{{{\mathrm{BA}}}}} = 1.64T_{{{\mathrm{W}}}}$. For the materials Sr_2_RuO_4_ and V_2_O_3_ studied in ref. ^2^, Katanin’s CW extraction of $\theta$-values implies $\tilde T_{{{\mathrm{K}}}} = 574\,{{{\mathrm{K}}}}$ or $164\,{{{\mathrm{K}}}}$, respectively. This illustrates, yet again, the main point of this reply: the Kondo scale is generically much smaller than $T_{{{{\mathrm{sp}}}}}^{{{{\mathrm{onset}}}}}$, and it is misleading to conflate these two scales.

## Supplementary information

Supplementary Information

## Data Availability

The authors declare that the data supporting the findings of this study are available from the authors.
