# Author Correction: Reply to: Extracting Kondo temperature of strongly-correlated systems from the inverse local magnetic susceptibility

**DOI:** 10.1038/s41467-023-35946-x

**Published:** 2023-01-13

**Authors:** Xiaoyu Deng, Katharina M. Stadler, Kristjan Haule, Seung-Sup B. Lee, Andreas Weichselbaum, Jan von Delft, Gabriel Kotliar

**Affiliations:** 1 Department of Physics and Astronomy, Rutgers University, Piscataway, NJ USA; 2 Physics Department, Arnold Sommerfeld Center for Theoretical Physics and Center for NanoScience, Ludwig-Maximilians-Universitat München, München, Germany; 3 Condensed Matter Physics and Materials Science Department, Brookhaven National Laboratory, Upton, NY USA

**Keywords:** Electronic structure, Electronic properties and materials, Theoretical physics

Correction to: *Nature Communications* 10.1038/s41467-021-21643-0, published online 04 March 2021

In the Acknowledgements section of this article the grant number relating to NSF DMR given for authors X.D. and G.K. was incorrectly given as 173307 and should have been 1733071. The original article has been corrected.

