# Supplementary material for: Reply to: Extracting Kondo temperature of strongly-correlated systems from the inverse local magnetic susceptibility
Source: Nat Commun. 2021 Mar 4;12:1445. doi: 10.1038/s41467-021-21643-0 (PMC7933365; doi:10.1038/s41467-021-21643-0)
Supplement: Supplementary file 1 — Supplementary Information [file 41467_2021_21643_MOESM1_ESM.pdf]

Supplementary material: Reply to "Comment on the signature of Mottness and Hundness in archetypal Correlated Metals" by A. Katanin

The Curie-Weiss law, $\chi(T) = \mu/(T+\theta)$, is often used to roughly characterize the temperature dependence of the susceptibility of Kondo impurities and of the local susceptibility of Hund metals. Correspondingly, a plot of $1/\chi(T)$ vs.T should give a straight line with slope $1/\mu$ and y-axis intercept θ/μ. Here we briefly elaborate on the extent to which this is the case for the data which we had published in [R2].

In our publication, we had computed $\chi_0^{\mathrm{sp}}(T)$, defined there as the static ($\omega \to 0$ limit of the dynamic) local spin susceptibility, for two setups: (i) realistic LDA+DMFT calculations for the material systems V_2O_3 and Sr_2RuO_4; and (ii) DMFT+NRG calculations for a Mott system M1 and a Hund system H1 in the context of a multi-orbital Hund-Hubbard model, which offers a minimal description of the physics of Hund's metals, and allows us to extract more precise values of susceptibility over several decades in temperature . In his comment, Katanin plotted $1/\chi_0^{\mathrm{sp}}(T)$ vs.T using our data from (i). In panels (a,b) of Supplementary Figure 1 below, we show similar plots obtained using our data from (ii). In both Katanin's plots and ours, the resulting curves can be fit fairly well by a "global" straight line $y(T) = (T+\theta_{\mathrm{glo}})/\mu_{\mathrm{glo}}$, but deviations are clearly discernable.

To quantify the extent to which a global Curie-Weiss description is too simplistic, we perform local analysis, viewing μ and θ not as constants, but as T-dependent parameters characterizing straight lines tangent to the inverse susceptibility curve. Concretely, for each value of T, we define the function $y(T') = [T' + \theta(T)]/\mu(T)$ as the straight line which is tangent to the curve $1/\chi_0^{\mathrm{sp}}(T')$ at $T' = T$, i.e. the latter curve has "local" slope $1/\mu(T)$, and its tangent crosses the y axis at $\theta(T)/\mu(T)$. Hence we define the temperature dependent moment and Curie Weiss temperature as $1/\mu(T) = d(1/\chi)/dT$, and $\theta(T)/\mu(T) = 1/\chi - T/\mu(T)$.

Panels c,d of Supplementary Figure 1 show the functions $\mu(T)$ and $\theta(T)$ so obtained for the Mott system M1 and the Hund system H1. They are not constant, indicating the limitations of a pure Curie-Weiss description.

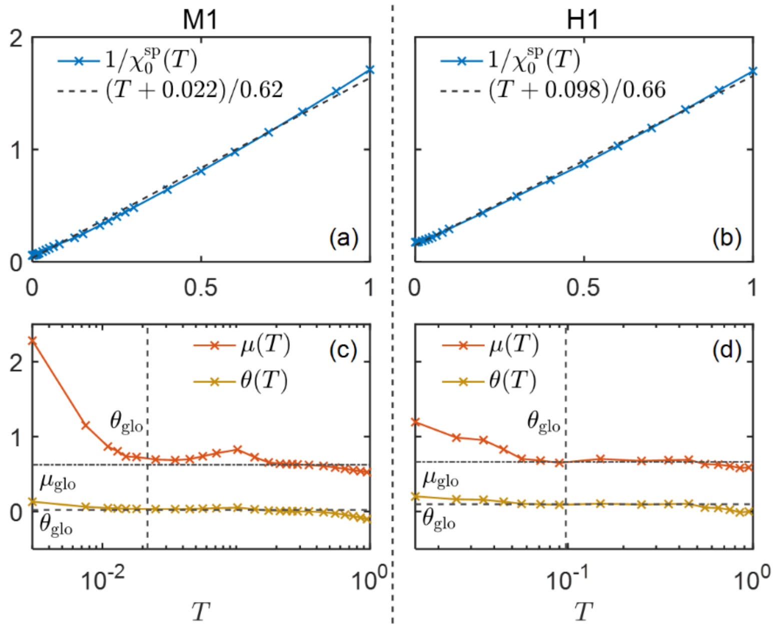

Supplementary Figure 1: Curie-Weiss analysis of the temperature dependence of the static local spin susceptibility $\chi_0^{\mathrm{sp}}(T)$, obtained from DMFT+NRG calculations [R2] for (a,c) a Mott system (M1; U=6.5, J=1) and (b,d) a Hund system (H1; U=3, J=1). (a,b) Global Curie-Weiss fits. (c,d) The functions $\mu(T)$ and $\theta(T)$ characterizing straight lines that are tangent to the inverse susceptibility (see text). The parameters μ_{glo} and θ_{glo} obtained from (a,b) are indicated in (c,d) by dashed-dotted or dashed lines, respectively.

The slight maximum in $\mu(T)$ for M1 in panel (c), near $T_{\mathrm{max}} \simeq 0.1$, reflects a slight decrease in the slope of $1/\chi_0^{\mathrm{sp}}(T)$ seen at that temperature in panel (a), which is also mentioned by Katanin in [R1] for the material system V_2O_3. Note that T_{max} it is about 5 times larger than the Curie-Weiss scale θ_{glo}. It reflects physics beyond that of a simple impurity model, requiring DMFT self-consistency instead, namely T_{max} is the onset of metallicity in the Mott system as the temperature is lowered. Indeed, the value of T_{max} agrees, up to a factor of two, with the temperature $T_{\mathrm{M}} = T_{\mathrm{spin}}^{\mathrm{onset}} \simeq 0.2$ which we had identified for the Mott system M1 in our paper as the temperature where a coherence resonance emerges from the pseudogap as the temperature is lowered.

Supplementary Figure 1 thus provides additional evidence supporting our assertion that for Mott systems, the onset of the Kondo resonance is not connected to logarithmic singularities, but instead is driven by the DMFT-self-consistency condition, as is well known in the DMFT literature.
